# Flavin Adenine Dinucleotide Rescues the Phenotype of Frataxin Deficiency

**DOI:** 10.1371/journal.pone.0008872

**Published:** 2010-01-25

**Authors:** Pilar Gonzalez-Cabo, Sheila Ros, Francesc Palau

**Affiliations:** 1 Laboratory of Genetics and Molecular Medicine, Instituto de Biomedicina de Valencia, CSIC, Valencia, Spain; 2 CIBER de Enfermedades Raras (CIBERER), Valencia, Spain; Hospital Vall d'Hebron, Spain

## Abstract

**Background:**

Friedreich ataxia is a neurodegenerative disease caused by the lack of frataxin, a mitochondrial protein. We previously demonstrated that frataxin interacts with complex II subunits of the electronic transport chain (ETC) and putative electronic transfer flavoproteins, suggesting that frataxin could participate in the oxidative phosphorylation.

**Methods and Findings:**

Here we have investigated the effect of riboflavin and its cofactors flavin adenine dinucleotide (FAD) and flavin mononucleotide (FMN) in *Saccharomyces cerevisiae* and *Caenorhabditis elegans* models of frataxin deficiency. We used a *S. cerevisiae* strain deleted for the *yfh1* gene obtained by homologous recombination and we assessed growth in fermentable and non-fermentable cultures supplemented with either riboflavin or its derivates. Experiments with *C. elegans* were performed in transient knock-down worms (*frh-1*[RNAi]) generated by microinjection of dsRNA *frh-1* into the gonads of young worms. We observed that FAD rescues the phenotype of both defective organisms. We show that cell growth and enzymatic activities of the ETC complexes and ATP production of *yfh1Δ* cells were improved by FAD supplementation. Moreover, FAD also improved lifespan and other physiological parameters in the *C. elegans* knock-down model for frataxin.

**Conclusions/Significance:**

We propose that rescue of frataxin deficiency by FAD supplementation could be explained by an improvement in mitochondrial respiration. We suggest that riboflavin may be useful in the treatment of Friedreich ataxia.

## Introduction

Friedreich ataxia (FRDA) is an autosomal recessive neurodegenerative disorder characterized by early onset and progressive limb and gait ataxia, dysarthria, deep tendon areflexia especially of the lower extremities, and presence of a sensory axonal neuropathy with motor conduction velocities greater than 40 m/s. In addition, most patients show hypertrophic cardiomyopathy. Additional non-neurological features are skeletal deformities and glucose intolerance or diabetes mellitus [1], [2]. The disease is caused by GAA triplet expansions [3] and point mutations [4], [5] in the *FXN* gene mapped to human chromosome 9q13. *FXN* encodes frataxin, a small protein of 210 amino acids expressed in the mitochondrial matrix [6]–[8]. Frataxin seems to act as a iron donor to other proteins for their utilization in different biochemical pathways, such as biogenesis of iron-sulfur clusters (ISC) [9]–[11] and activation of aconitase [12]. Thus, the pathogenic consequences of frataxin deficiency have been related with defects of ISC biogenesis but also with iron deposits [13], oxidative stress [8] and regulation of the mitochondrial respiratory chain [14], [15].

Based on cell and mitochondrial effects of the lack of frataxin, several pharmacological approaches have been proposed. These include the use of antioxidants to reduce radical oxidative species. Idebenone is a synthetic analogue of ubiquinone or coenzyme Q_10_ (CoQ), which has antioxidant activity and is able to act in situations of low concentrations of oxygen. It has the ability to inhibit the lipidic peroxidation, protecting the cellular membranes and the mitochondria from oxidative damage. It is able to stimulate the mitochondrial functions and increase the energetic contribution to the myocardium. A number of clinical trials have been conducted, suggesting a protecting effect on the cardiac hypertrophy [16]–[21]. More recently, some beneficial effects of idebenone on the neurological symptoms have also been described [22], [23]. Mitoquinone (MitoQ) is another proposed CoQ derivative with antioxidant activity selectively directed to the mitochondria [24], [25].

Alternative pharmacological strategies have been used. Iron chelators mobilize the iron deposits observed in patients [26]. Chelator treatment with deferiprone causes no apparent hematologic or neurologic side effects while reducing neuropathy and ataxic gait in the youngest patients [27].

Expansion of the GAA trinucleotide reduces transcription of *FXN* gene, which in turn leads to frataxin deficiency. To reverse *FXN* silencing, a class of histone deacetylase (HDAC) inhibitors have been proposed as an alternative therapy [28]–[30]. Recently, treatment of a FRDA mouse model by an HDAC inhibitor compound has shown correction of biological parameters of frataxin deficiency [31].

Some data suggest that frataxin may be involved in the energetic metabolism. Clinical studies applying magnetic resonance show a failure in the production of ATP in the patients' muscles [32]. Overexpression of human frataxin in human adipocytes increases the activity of the electron transport chain, mitochondrial membrane potential and ATP production [14]. By genetic and biochemical analyses in *Saccharomyces cerevisiae* we have demonstrated a physical and functional interaction among the yeast frataxin, Yfh1p, and succinate dehydrogenase subunits Sdh1p and Sdh2p of complex II of the respiratory chain [15]. We have also confirmed the interaction among frataxin and both SDHA and SDHB human proteins. Additionally, we also demonstrated genetic interaction between frataxin and SDHC subunit in *Caenorhabditis elegans* [33]. All these data point to a direct role of frataxin on the complex II of the electronic transport chain (ETC); thus, lack of frataxin may induce a failure in the oxidative phosphorylation (OXPHOS) by means of abnormal function of the electron transport at complex II.

Transport of electrons through the ETC is needed to correct reduction of CoQ. In mammalian cells electrons are provided to CoQ not just by reduction of complex I and complex II but also by the electron transfer flavoprotein (ETF) complex, a system composed by the ETF-dehydrogenase (ETF-QO) and ETF, a heterodimer composed by two subunits (ETFα and ETFβ), that delivers electrons coming from β-oxidation of fatty acids and amino acid catabolism to CoQ [34]. In *S. cerevisiae* β-oxidation occurs mainly in peroxisomes but not in mitochondria; however, homologous genes for ETF complex genes have been reported in yeast: *ypr004c* as the *ETFα* homologue and *ygr207c* as the homologue of *ETFβ.* We have also demonstrated that Yfh1p interacts with two of these components of the electron transfer flavoprotein complex [15].

Human complex II is a multimeric enzyme composed of four subunits: a flavoprotein (SDHA), an iron-sulphur subunit (SDHB), and two proteins (SDHC and SDHD) that is anchored to the inner mitochondrial membrane. Complex II carries electrons to the ubiquinone pool and constitutes the second essential oxidation–reduction reaction of the respiratory chain. Moreover, SDHA and SDHB subunits compose the active enzyme succinate dehydrogenase that oxidizes succinate to fumarate in the Krebs cycle [35]. Complex II has flavin adenine dinucleotide (FAD) as a prosthetic group that is covalently anchored to the SDHA subunit. FAD also acts as a cofactor to the ETF complex. FAD and flavin mononucleotide (FMN) are cofactors derived from riboflavin, a water-soluble vitamin that have been used in the treatment of several mitochondrial disorders such as complex I deficiency [36], short-chain acyl coenzyme A dehydrogenase (SCAD) [37], mitochondrial encephalomyopathy with lactic acidosis and stroke-like episodes (MELAS) syndrome [38], L-2-hydroxyglutaric aciduria [39], and in complex II deficiency [40].

Based on our finding that frataxin interacts with subunits of the complex II and with components of the electron transfer flavoprotein complex we wonder whether riboflavin could be useful in the treatment of FRDA as well. To address this question we investigated the effect of riboflavin and riboflavin-derived cofactors on frataxin-deficient strains of *S. cerevisiae* and *C. elegans*. Our results show that the flavin adenin dinucleotide is able to rescue the phenotype of both mutant organisms but, this improvement is not dependent of complex II activity.

## Results

### Riboflavin-Derived Cofactors Are Able to Improve the Growth of a Deficient yfh1Δ Strain

**Figure 1:**
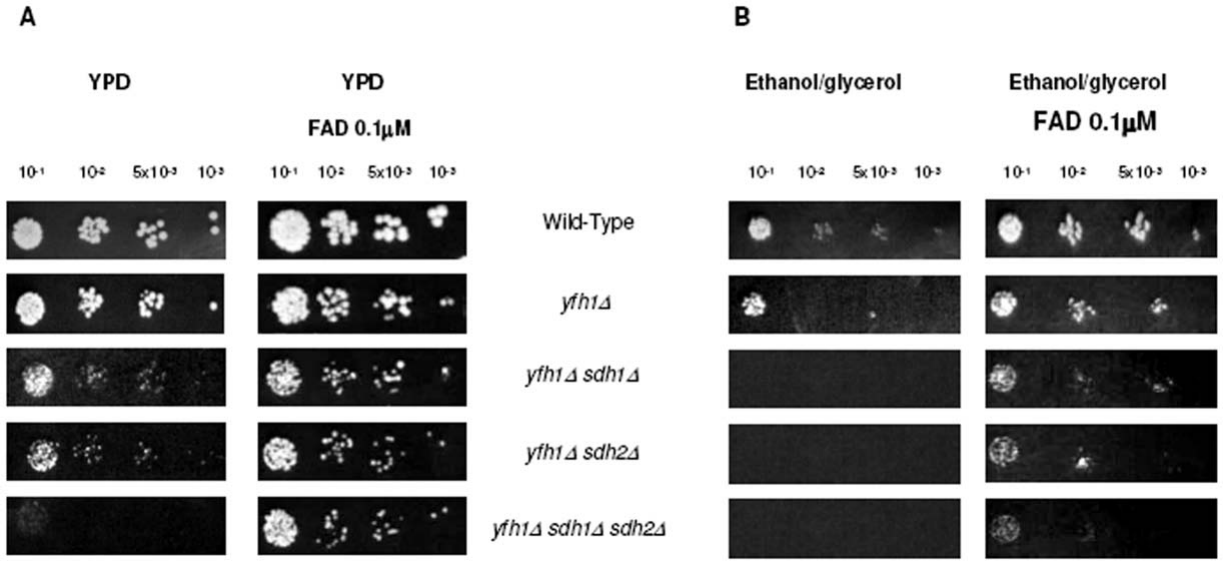
Analysis of the yeast growth. Serial dilutions of cell suspensions of the different strains were spotted on either YPD rich medium or ethanol/glycerol medium and incubated at 30°C for 48 hours. **A**) Cells grown on rich YPD plates with or without 0.1 µM FAD supplementation. **B**) Cells were grown on synthetic ethanol/glycerol medium plates with or without 0.1 µM FAD supplementation.

**Figure 2:**
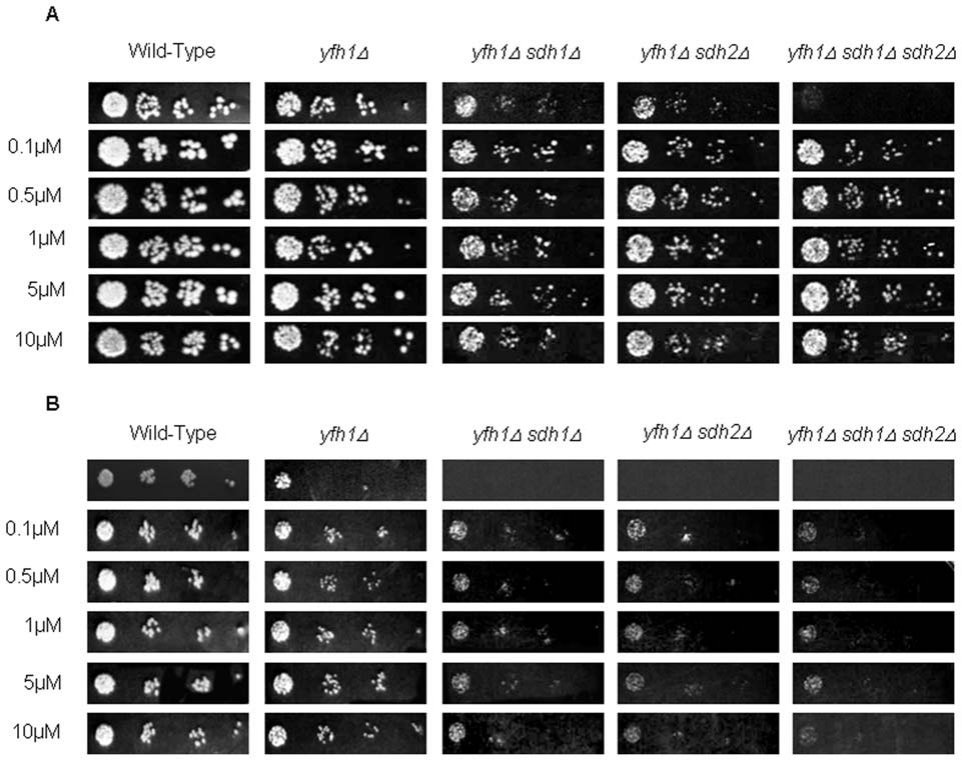
Dose-response analysis of FAD supplementation. **A**) Serial dilutions of cell suspensions of the different strains were spotted on YPD medium supplemented with increasing concentrations of FAD and incubated at 30°C for 48 hours. **B**) The wild-type and mutants' strains were also spotted in ethanol/glycerol medium supplemented with increasing concentrations of FAD and incubated at 30°C for 48 hours.

**Figure 3:**
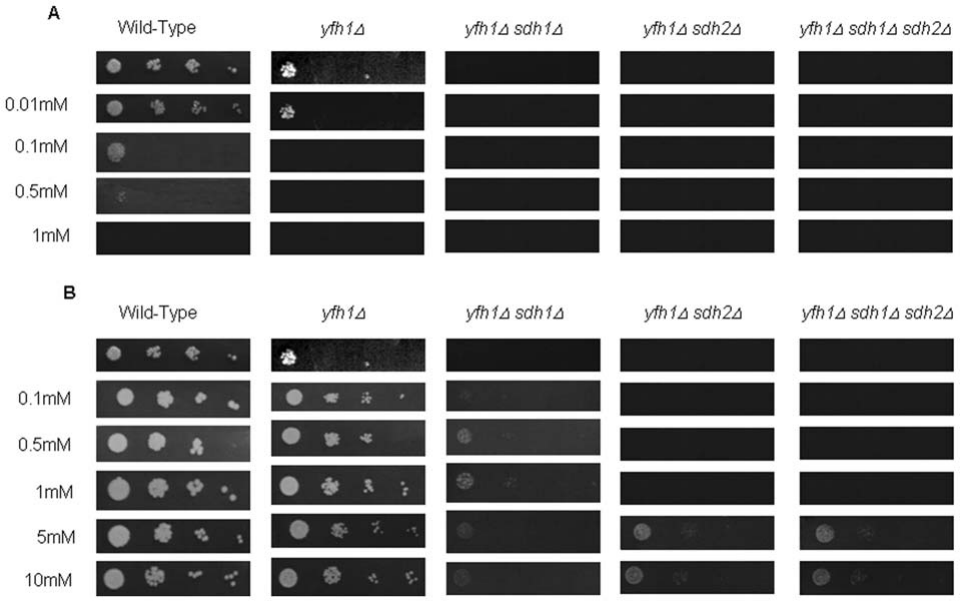
Dose-response analysis of riboflavin and FMN supplementation. Serial dilutions of cell suspensions of the different strains were spotted on in ethanol/glycerol medium supplemented with increasing concentrations of riboflavin (**A**) and FMN (**B**), and incubated at 30°C for 48 hours.

**Figure 4:**
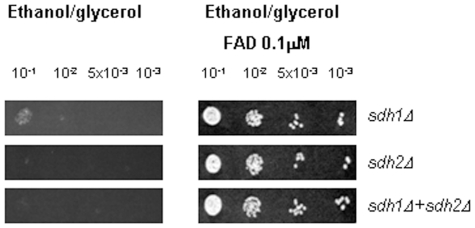
Analysis of the effect of FAD on *sdh1Δ* and *sdh2Δ* cells growth. Serial dilutions of cell suspensions of the different strains were spotted on the indicated media and incubated at 30°C for 48 hours. Cells were grown on synthetic ethanol/glycerol medium plates supplemented with 0.01 µM FAD.

We have previously described genetic and physical interaction among yfh1p, the yeast ortholog of mammalian *FXN*, and succinate dehydrogenase complex subunits Sdh1p and Sdh2p of the yeast mitochondrial ETC [15]. We have observed that the single mutant for yfh1p showed regular growth in fermentative conditions (YPD medium), whereas double mutants *yfh1Δ sdh1Δ* and *yfh1Δ sdh2Δ* showed a poor growth in such conditions, and the triple mutant, *yfh1Δ sdh1Δ sdh2Δ*, was even poorer (Figure 1A, left panel). When cells were cultured in respiratory conditions (ethanol/glycerol medium) all mutant strains including the single mutant showed abnormal growth (Figure 1B, left panel). As FAD is the cofactor of complex II we initially decided to investigate the effect of FAD in mutant yeast growth. First of all, we performed a dose-dependent study with FAD in yeast strains (Figure 2). We used a range of different doses, between 0.1 µM to 10 µM. We analyzed yeast growth in the yfh1Δ single mutant, the double mutants yfh1Δ sdh1Δ and yfh1Δ sdh2Δ, and the triple mutant yfh1Δ sdh1Δ sdh2Δ (see Supplementary Table S1), in both fermentative and respiratory conditions. Addition of FAD to cultures improved cell growth of single *yfh1Δ* and double mutants' *yfh1Δ sdh1Δ* and *yfh1Δ sdh2Δ* in fermentative conditions (Figure 1A, right panel). Interestingly, growth amelioration of the triple mutant *yfh1Δ sdh1Δ sdh2Δ* in the presence of FAD was outstanding (Figure 1A, right panel). Growth was also induced by FAD in respiratory conditions (Figure 1B, right panel). Double mutants and the triple mutant were not able to growth in ethanol/glycerol medium and *yfh1Δ* strain grew poorly, but in all cases the growth rate of yeast cells improved in the presence of FAD. These findings suggest that FAD may be able to rescue the growth of the strains in both fermentative and respiratory conditions. Then we checked whether abnormal growth in respiratory conditions could be rescued by addition of either riboflavin or FMN cofactor. Riboflavin did not improve the growth of any strain (Figure 3A). In fact, riboflavin seemed to be toxic at the concentrations we used. By contrast, cultures in the presence of FMN improved cell growth (Figure 3B), but using higher doses that those use for FAD. We wonder whether the effect of the FAD was through complex II. To answer this question we tested the growth of single mutants *sdh1Δ, sdh2Δ* and the double mutant *sdh1Δ sdh2Δ*. In these strains, FAD rescues the phenotype and growth perfectly in respiratory conditions (Figure 4). These results suggest that growth recovery is not dependent on the presence of complex II subunits Sdh1p and Sdh2p.

### FAD Cofactor Improves Activities of the ETC Complexes Except for Complex II

**Figure 5:**
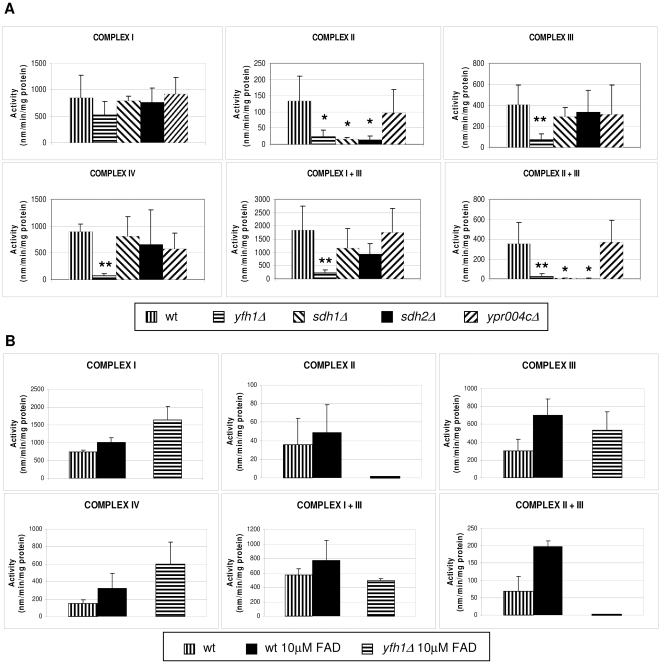
Enzymatic activity analysis of the ETC in yeast mitochondria. **A**) Wild-type, *yfh1*Δ, *sdh1*Δ, *sdh2*Δ, *ypr004c*Δ cells were grown in YPD media under aerobic conditions until exhaustion of the carbon source. Mitochondria were isolated and used to determine the enzymatic activity of the complex I, II, III, IV, I+II and I+III of the respiratory chain. Errors bars indicate the standard deviation of at least three independent measurements. (t-Student, *p<0.05; **p<0.01). **B**) Analysis of addition of FAD on ETC complexes activities. FAD increases the activity of ETC complexes. Wild-type and *yfh1*Δ cells were grown in ethanol/glycerol medium with 10 µM FAD in aerobic conditions. Mitochondria were isolated and used to determine the enzymatic activity of the complex I, II, III, IV, I+II and I+III of respiratory chain. No histogram is indicated for *yfh1*Δ cells with any FAD addition because they did not grow on ethanol/glycerol medium. Errors bars indicate the standard deviation of at least three independent assays.

To address whether this improvement is due to an effect on the respiratory chain activity, we measured the activity of complexes I, II, III and IV in a wild-type strain (W303) and in the single mutants *yfh1Δ*, *sdh1Δ*, *sdh2Δ*, and *ypr004cΔ* (the yeast homologue of the mammalian electron transfer flavoprotein α gene, *ETFα*). The frataxin mutant showed a significantly decreased activity in every complex except for complex I (Figure 5A). Both *sdh1Δ* and *sdh2Δ* strains showed abnormal activities for complex II and complexes II+III. As expected, the absence of *ypr004c* gene did not affect any enzymatic activity of the ETC. Thus, we proceeded to investigate the effect of FAD in ETC complexes activities in ethanol/glycerol medium that forces yeast to respire. Since the growth response of the mutant strain to FAD had not been dose dependent and ETC measures were performed on mitochondrial extracts from yeast cells grown in liquid medium, we decided to use the higher dose, 10 µM. We found an improvement of every complex activity in the wild-type. Interestingly, in the *yfh1Δ* mutant we observed a significant amelioration of complex I, complex III, complex IV and complex I+III activities but not in complex II and complex II+III activities (Figure 5B). Thus, FAD increases the activity in every complex except complex II. This result confirms the observation that FAD effect on growth improvement of frataxin-deficient cells is acting independently of complex II.

### FAD Cofactor Increases the Production of ATP in Frataxin Mutant

**Figure 6:**
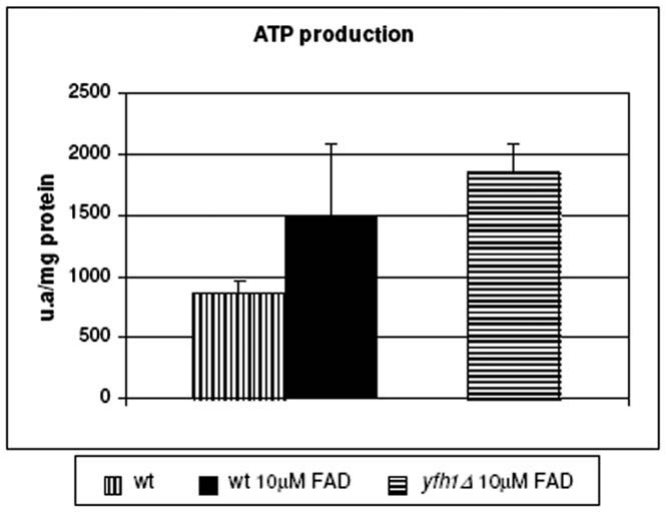
Analysis of the ATP synthesis in yeast mitochondria. ATP production was greater in cells grew with FAD. Wild-type and *yfh1*Δ cells were grown in ethanol/glycerol medium under aerobic conditions with or without 10 µM FAD. No histogram is indicated for *yfh1*Δ cells with any FAD addition because they did not grow on ethanol/glycerol medium. Mitochondria were isolated and used to determine ATP production using the Adenosine 5′-triphosphate (ATP) Bioluminescent Assay Kit as indicated by the manufacturer. Errors bars indicate the standard deviation of at least three independent assays.

To know the effect of improvement of ETC activities in the oxidative phosphorylation we measured ATP production in respiratory conditions. When adding FAD to wild-type cells we observed an increase of ATP production that correlated with increased activities of ETC complexes. In such conditions only wild-type cells showed a good growth; by contrast, yfh1Δ grew very poorly. However, addition of FAD not only induced cell growth but also increased ATP production even to higher levels that of wild-type yeast (Figure 6).

### Alternative Pathways for FAD Action

**Figure 7:**
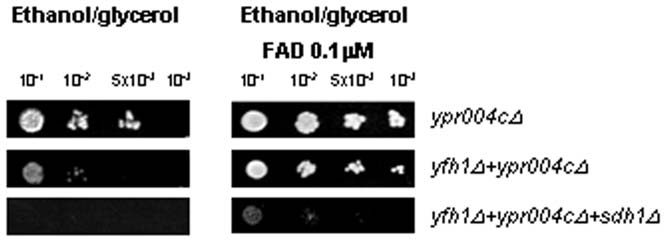
Analysis of the effect of FAD on *ypr004cΔ* cells growth. Serial dilutions of cell suspensions of the different strains were spotted on the indicated media and incubated at 30°C for 48 hours. Cells were grown on synthetic ethanol/glycerol medium plates supplemented with 0.01 µM FAD.

To know about the action of FAD on ETC and OXPHOS activities in an independent manner of complex II we investigate the effect of the cofactor on the Etf complex. We performed growth experiments under respiratory conditions in mutant strains for putative *Etfα* subunit, *ypr004c*, of the Etf complex (Figure 7). Although *ypr004cΔ* strain could grow under respiratory conditions it strongly ameliorated when FAD was added to the culture; *yfh1Δ ypr004cΔ* double mutant grew poorer than the single *ypr004cΔ* strain but it was also rescued by FAD, as we previously had observed for the *yfh1Δ sdh1Δ* and *yfh1Δ sdh2Δ* mutants. To know if the FAD effect depends on the presence of at least one of *yfh1*, *sdh* or *ypr004c* genes we analyzed yeast growth of the *yfh1Δ sdh1Δ ypr004cΔ* strain. The triple mutant strain did not grow under respiratory conditions but growth was partially rescued when adding FAD. Altogether, these findings suggest that the biological action of FAD on yeast growth do not exclusively depend on the presence of these three genes.

### FAD and FMN, but Not Riboflavin, Partially Rescue the Phenotype in Frataxin-Deficient Worms

**Figure 8:**
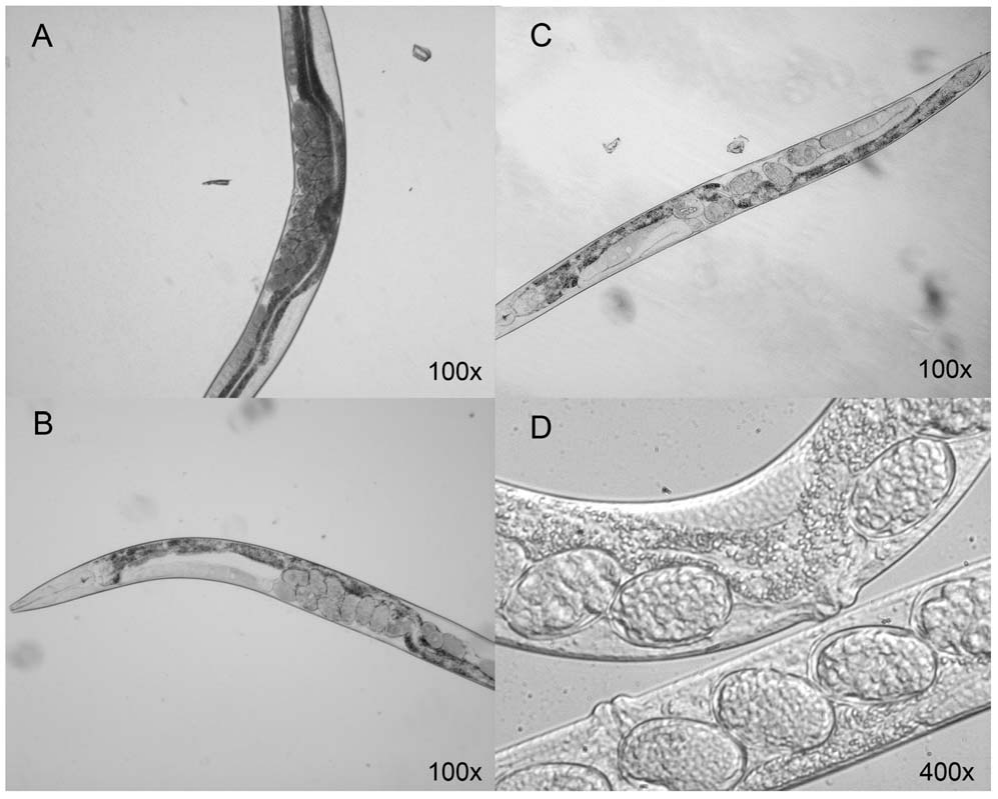
Phenotypic analysis of *frh-1*[RNAi] worms. Nomarski photographs show worms of 5 days-old cultured at 20°C. **A**) *cat*[RNAi] worm as a control showing normal morphology. **B**) *frh-1*[RNAi] worm showing thinner morphology, pale body and fewer eggs into the worm. **C**) *frh-1*[RNAi] worm incubated in NGM agar supplemented with 0.5 mM FMN. Treated frh-1[RNAi] worms show increase in the number of eggs when comparing with not treated knock-down worms (figure 6B). We observed general improvement in the phenotype. **D**) Comparison of the morphology of *frh-1*[RNAi] worms supplemented with 5 µM FAD (top) or with no drug supplementation (bottom); the worm grown in the supplemented medium was less thin and morphology was improved.

The previous results clearly show that both FAD and FMN rescue the deficiency of frataxin in *S. cerevisiae*. To find out if these flavins could be developed as potential therapeutic agents, it would be interesting to show that they can also act in the same fashion in a multicellular organism such us *C. elegans*. Experiments were performed in transient knock-down worms (*frh-1*[RNAi]) generated by microinjection of dsRNA *frh-1* into gonads of adult worms. The F1 progeny of injected nematodes show a pleiotropic phenotype when compared with controls (Figure 8A) which includes pale body colour and reduced number of eggs within the worm (Figure 8B), thin morphology, reduced lifespan, reduced brood size, altered defecation, and increased sensitivity to oxidative stress [33].

We analyzed morphological appearance and physiological parameters in five-day-old *frh-1*[RNAi] worms growing in plates with riboflavin or riboflavin-derived cofactors. We observed an evident increase in the worm size with either FAD or FMN. Worms seemed to be healthier, to lay more eggs and to improve the egg development (Figures 8C and 8D). Worms supplemented with FAD improved their fertility as well as we observed increased number of eggs and larvae compared with *frh-1*[RNAi] control worms (data not shown). By constrast, riboflavin addition did not improve the knock-down phenotype.

### FAD and FMN Increase Lifespan, Fertility and Defecation Rhythm in Knock-Down Worms

**Figure 9:**
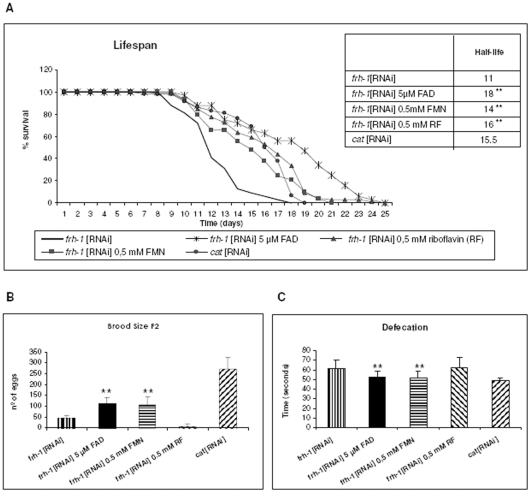
Lifespan and other physiological parameters analyses of frh-1[RNAi] nematodes. **A**) Worms were transferred into fresh NGM plates supplemented with FAD, FMN or riboflavin at 20°C from larval stage L1 until they died. These worms were compared with *frh-1*[RNAi] worms grew without cofactors and with *cat*[RNAi] worms as a negative control. **B**) Proportion of dead embryos was determined in *frh-1*[RNAi] worms treated with FAD, FMN and riboflavin at 20°C. Five worms were analyzed for each drug. These worms were compared with *frh-1*[RNAi] worms grew without cofactors and with *cat*[RNAi] worm as a negative control. Histograms show means ± SEM. **C**) Analysis of defecation in frataxin deficient *frh-1*[RNAi] worms. Defecation interval represents the mean between two pBoc contractions and shows significant differences among *frh-1*[RNAi] worms and *frh-1*[RNAi] worms supplemented with FAD (p<0.01) and FMN (p<0.01). *cat*[RNAi] worm as a negative control. Histograms show means ± SEM.

*frh-1*[RNAi] worms show a pleiotropic phenotype and physiological behaviour that include reduced longevity, uncoordinated pharynx pumping, egg laying defects (Egl phenotype), lower number of eggs within the uterus, reduced brood size and abnormal defecation rate [33]. Thus, we investigated the effect of flavins in the lifespan, egg laying and defecation of frh*-1*[RNAi] nematodes. Lifespan was determined by counting the number of days from the first larval stage (L1) until the worm died. We analyzed approximately 40 worms for each drug, and the experiment was performed twice. Lifespan is significantly recovered in all conditions (FAD, p<0.01; FMN, p<0.01; riboflavin, p<0.01) (Figure 9A). Half lifespan for control *cat*[RNAi] worms was 15.5±2.9 days, and decreased to 11±2.3 days for *frh1*[RNAi] individuals. FAD increased half lifespan to 18±4.6 days, that is, seven days more than the frataxin-deficient worms, whereas the increased lifespan with either FMN or riboflavin was lower but still significant.

To study fertility we counted the eggs laid by each worm in individual plates and moved to fresh plates every eight hours. We observed that brood size was recovered after addition of with FAD (p = 0.01) or FMN (p = 0.01) (Figure 9B), although values did not reach to those of control worms. This effect agrees with the apparent observation of the egl-1 phenotype rescue we have observed in the *frh-1*[RNAi] worms after treatment.

Defecation in C. *elegans* is achieved by periodically activating a stereotyped sequence of muscle contractions. Each defecation cycle can be thought of as having three distinct steps: the posterior body muscle contraction (pBoc), the anterior body muscle contraction (aBoc), and the expulsion (Exp), which consists of the intestinal muscle and anal depressor contractions. Each of these three steps appears to be controlled by a separate set of motor neurons, and all three steps are co-ordinately and cyclically activated from some unidentified source [41]. Defecation in *frh-1*[RNAi] worms is altered with increasing the rhythm of defecation between consecutive pBoc contractions [33]. We measured the interval time from pBoc to the next pBoc for 10 consecutive cycles in 10 worms, comparing *frh-1*[RNAi] worms treated with flavins and *frh-1*[RNAi] worms with no treatment. We observed significant recovering of defecation when adding flavins to cultures (FAD, p<0.01; FMN p<0.01) (Figure 9C).

## Discussion

We had previously showed that both yeast and human frataxins interact with complex II subunits Sdh1p/Sdh2p and SDHA/SDHB, respectively [15]. FAD is the prosthetic cofactor of complex II covalently bound to the flavoprotein subunit Sdh1p/SDHA. FAD and FMN are cofactors derived from the metabolism of riboflavin, which has been employed in the treatment of several disorders involving different enzymatic complexes of the OXPHOS system: complex I deficiency [36], [42], [43], complex II deficient patients, at least by reducing the rate of disease progression [40], and in a boy with Leigh syndrome and complex II deficiency [44]. Response to riboflavin in patients with defects of either β-oxidation [45], [46] or single flavo-apoenzymes: pyruvate dehydrogenase [47], electron transfer flavoprotein [48] and short-chain acyl coenzyme A dehydrogenase [37] have been reported as well. Riboflavin has also been used for the treatment of respiratory chain disorders in combination with other cofactors such as nicotinamide in patients with MELAS syndrome, having a favourable response [49].

Thus, we hypothesized that may riboflavin and its cofactors FAD and FMN might be good candidate drugs for FRDA therapy. To address this point we have investigated the effect of riboflavin, FAD and FMN in two organism models of frataxin deficiency, *S. cerevisiae* and *C. elegans*. In both organisms we could partially rescue the abnormal phenotype. This pharmacological effect was more evident for FAD. We have observed that FAD improves growth of *yfh1Δ* yeast in respiratory conditions and several physiological parameters of *frh-1*[RNAi] knock-down worms, such as lifespan, fertility and defecation. We also observed some amelioration by FMN although it was milder than FAD. Riboflavin showed contradictory results: it was toxic in yeast at the concentrations we used but lifespan was increased in the knock-down worms. Specific metabolic routes for riboflavin have been described in both organisms [50], so we suspect that such a toxic effect in yeast may be dose-dependent [51].

Riboflavin provides FMN and FAD, which function as coenzymes in respiratory chain complexes I and II, respectively, but the mechanism underlying its possible efficacy in respiratory chain disorders is still poorly understood. Different arguments have been proposed to explain the beneficial of riboflavin treatment. Riboflavin may act by inhibiting the proteolytic breakdown of complex I, with a subsequent increase in enzymatic activity [52], [53]. On the other hand, riboflavin supplementation could exert a general stabilizing effect on the assembly and correct functioning of mitochondrial flavin-dependent complexes I and II, generating a positive functional effect on the catalytic activity.

In the case of frataxin deficiency we hypothesized that riboflavin may provide FAD molecules to complex II improving energetic metabolism by addressing electrons towards CoQ. However, while growth recovery of *yfh1*Δ could be explained by this way growth recovery of both *Sdh1*Δ and *Sdh2*Δ strains does not agree with such a postulate as the appropriate structure of complex II in these mutants is defective. The observed growth amelioration of these mutant yeast strains should depend on other biochemical routes. Since both ETF heterodimer and ETF-QO have FAD as a prosthetic group we hypothesized that ETF complex may be a candidate for FAD action. ETF serves as an electron acceptor for the acyl-CoA dehydrogenases involved in fatty acid oxidation as well as for several dehydrogenases involved in amino acid and choline metabolism. Subsequently, these electrons are transferred via ETF-QO to CoQ in the respiratory chain. Thus, we introduced the putative yeast gene for *Etfα*, *ypr004c*, in our analysis. Yeast cells defective for *ypr004c* showed almost normal growth in respiratory conditions and normal enzymatic activities of ETC complexes in fermentative conditions. Single mutants for *yfh1*, *sdh1*, *sdh2* or *ypr004c* responded to FAD when growing cells in respiratory conditions. Even double mutants of *yfh1* with *sdh1*, *sdh2* or *ypr004c* respond to FAD as well. To observe much reduced response to FAD all three genes, *yfh1*, *sdh1* and *ypr004c* should be deleted in yeast cells but in such conditions some growth is still observed. These results may be interpret as FAD is acting via complex II and Etf complex but at least another route still remain relevant. This route may be via complex I that remain unaffected in yeast cells defective for *yfh1*. Supporting this is the fact that FAD strongly increase complex I activity of the *yfh1Δ* cells in respiratory conditions. Based on our results we suggest that riboflavin may be useful in the treatment of Friedreich ataxia patients as in other OXPHOS disorders.

## Materials and Methods

### Strains

Strains used in this study are listed in Supplementary Material, Table S1.

### Phenotypic Analyses in Yeast

The effect of riboflavin and riboflavin derivatives (FMN and FAD) on mutants was assessed by growing the strains in rich (YPD; yeast extract, peptone, dextrose) and ethanol-glycerol medium. Previously, cells growing exponentially in YPD medium were harvested and adjusted to 0.1 units of absorbance at 600 nm. Serial dilutions were made with sterile water and 3 µl of each dilution was spotted on the different culture media and different riboflavin concentrations (0.01 mM, 0.1 mM, 0.5 mM, 1 mM), FMN (0.1 mM, 0.5 mM, 1 mM, 5 mM, 10 mM) and FAD (0.1 µM, 0.5 µM, 1 µM, 5 µM, 10 µM). Plates were incubated at 30°C for 48 hours.

### Obtaining Crude Mitochondria

Wild-type, *yfh1Δ, sdh1Δ, sdh2Δ* and *ypr004cΔ* strains were grown in rich medium (YPD; yeast extract, peptone, dextrose). Wild-type and *yfh1*Δ cells were grown in ethanol/glycerol medium with 10 µM FAD in aerobic conditions and only wild-type in ethanol/glycerol medium without FAD. Isolation of yeast mitochondria was as described [54], and were used for assays of ETC enzymatic activities.

### Mitochondrial Respiratory Chain Activities

Freshly obtained mitochondria were assayed for complex I, complex II, complex III, complex IV, complex I+II and complex II+III activities. All assays were performed in triplicate at 30°C with stirring. In all the reactions mitochondrial protein (10–60 µg) was incubated in reaction buffer (40 mM sodium phosphate, pH 7.4) for 1 min.

#### Complex I

The reaction was initiated with 75 µM DCIP, without NADH; after 1 min, 200 µM NADH was added. Specific activity of DCIP reduction was determined at 600 nm with an extinction coefficient of 19.1 mM^−1^ cm^−1^.

#### Complex II

The reaction was initiated with the addition of 250 µM potassium cyanide and 75 µM DCIP; after 1 min, 40 mM sodium succinate was added. Reactions were monitored via spectrophotometric measurements of absorbance at 600 nm. The specific activity was determined with an extinction coefficient of 19.1 mM^−1^ cm^−1^.

#### Complex III

The reaction was initiated with the addition of 250 µM potassium cyanide and 100 µM cytochrome *c*; after 1 min, 50 µM decylubiquinone was added. The reaction was monitored via spectrophotometric measurements of absorbance at 550 nm. Specific activity was determined with an extinction coefficient of 19.5 mM^−1^ cm^−1^.

#### Complex IV

The reaction was initiated with the addition of 100 µM cytochrome *c* reduce with ascorbate. The reaction was monitored via spectrophotometric measurements of absorbance at 550 nm. Specific activity was determined with an extinction coefficient of 19.5 mM^−1^ cm^−1^.

#### Complex I+III

The reaction was initiated with the addition of 250 µM potassium cyanide and 100 µM cytochrome *c*; after 1 min, 100 µM NADH was added. The reaction was monitored via spectrophotometric measurements of absorbance at 550 nm. Specific activity was determined with an extinction coefficient of 19.5 mM^−1^ cm^−1^.

#### Complex II+III

The reaction was initiated with the addition of 250 µM potassium cyanide and 100 µM cytochrome c; after 1 min, 40 mM sodium succinate was added. The reaction was monitored via spectrophotometric measurements of absorbance at 550 nm. Specific activity was determined with an extinction coefficient of 19.5 mM^−1^ cm^−1^.

### ATP Assays

Mitochondrial concentrations of ATP were determined using the Adenosine 5′-triphosphate (ATP) Bioluminescent Assay Kit (Sigma) following the manufacturer's instructions.

### Strain and Worm Culture

We used the *C. elegans* strain Bristol N2, which was supplied by the *Caenorhabditis* Genetics Centre (University of Minnesota, MN). Worms were maintained on nematode growth medium (NGM) with *Escherichia coli* strain OP50 [55] and supplemented with riboflavin or its cofactors when appropriate. Standard supplementation experiments were performed with 0.5 mM riboflavin, 5 µM FAD or 0.5 mM FMN. Worms were incubated at 20°C and the progeny was analyzed.

### RNA Interference

RNAi in *C. elegans* was performed as described previously elsewhere [33].

### Phenotypic and Physiological Assays in *C. elegans*

We compared *frh-1*[RNAi] worms treated with drugs and *frh-1*[RNAi] worms without drugs by analyzing brood size of F2, lifespan and defecation. We observed the morphology under optic microscope of worms of five years old after hatching. They were immobilized with 10 mM levamisole and were mounted on pads with M9 buffer to avoid desiccation. To determine the lifespan, approximately 40 hermaphrodite worms were transferred into fresh NGM plates at 20°C until they died. Nematodes were transferred into new plates during the experiments to avoid mixing tested worms with their offspring. The viability of adult animals was checked under a stereomicroscope everyday and when they did not move after touching with a platinum wire they were considered as dead. The size of F2 offspring was assessed by placing a single worm on individual NGM plates supplemented with drugs at 20°C, moving it every eight hours and counting the eggs laid. Five worms were analyzed for each drug. Defecation cycles were analyzed by observation of L4 larvae under the stereomicroscope, measuring the time from pBoc to next pBoc. For each drug, ten worms were followed for ten continuous defecation cycles at 20°C.

### Statistical Analysis

Data are expressed as mean ± SEM. For lifespan analysis, data were compared by log rank Mantel-Cox test statistical analysis. The Mann-Whitney test was used for statistical analysis of F2 brood-size and defecation. Statistic significance for enzymatic activities of the mitochondrial respiratory chain complexes was determined by Student's t test. p<0.05 was considered significant.

## Supporting Information

Table S1 *Saccharomyces cerevisiae* strains used in this study
(0.06 MB DOC)
