# Supplementary material for: Flavin Adenine Dinucleotide Rescues the Phenotype of Frataxin Deficiency
Source: PLoS One. 2010 Jan 25;5(1):e8872. doi: 10.1371/journal.pone.0008872 (PMC2810331; doi:10.1371/journal.pone.0008872)
Supplement: Table S1 — Saccharomyces cerevisiae strains used in this study (0.06 MB DOC) [file pone.0008872.s001.doc]

**SUPPLEMENTAL DATA**

Supplemental Data include 1 table.

| Strain | Genotype | Reference |
| --- | --- | --- |
| W303-1A | *MATa ade2 ura3 his3 trp1 leu3 can1* | (1) |
| W303-1B | *MATα ade2 ura3 his3 trp1 leu3 can1* | (1) |
| *yfh1Δ* | *yfh1Δ::KanMx* derivative of W303-A | (2) |
| *sdh1Δ* | *sdh1Δ::LEU2* derivative of W303-B | (3) |
| *sdh2Δ* | *sdh2Δ::TRP1* derivative of W303-B | (3) |
| *ypr004cΔ* | *ypr004c::URA3* derivative of W303 | (3) |
| *yfh1Δ sdh1Δ* | *yfh1Δ:: KanMx sdh1Δ::LEU2* derivative of W303 | (3) |
| *yfh1Δ sdh2Δ* | *yfh1Δ:: KanMx sdh2Δ::TRP1* derivative of W303 | (3) |
| *sdh1Δ sdh2Δ* | *sdh1Δ::LEU2 sdh2Δ::TRP1* derivative of W303 | (3) |
| *yfh1Δ ypr004cΔ* | *yfh1Δ:: KanMx ypr004c::URA3* derivative of W303 | (3) |
| *yfh1Δ sdh1Δ sdh2Δ* | *yfh1Δ:: KanMx sdh1Δ::LEU2 sdh2Δ::TRP1* derivative of W303 | (3) |
| *yfh1Δ sdh1Δ ypr004cΔ* | *yfh1Δ::KanMx sdh1Δ::LEU2 ypr004c::URA3* derivative of W303 | (3) |

**Table S1:** *Saccharomyces cerevisiae* strains used in this study.

**REFERENCES**

1. Thomas, B. J., and Rothstein, R. (1989) *Cell* 56(4), 619-630

2. Rodriguez-Manzaneque, M. T., Tamarit, J., Belli, G., Ros, J., and Herrero, E. (2002) *Mol Biol Cell* 13(4), 1109-1121

3. Gonzalez-Cabo, P., Vazquez-Manrique, R. P., Garcia-Gimeno, M. A., Sanz, P., and Palau, F. (2005) *Hum Mol Genet* 14(15), 2091-2098
